# The relationship between gene positive hypertrophic cardiomyopathy patients and presence of scar on cardiac magnetic resonance imaging

**DOI:** 10.1186/1532-429X-16-S1-P339

**Published:** 2014-01-16

**Authors:** Jacob Blount, Andrew Sumner, Laurie A Fleming, Mercedes Scott, Matthew W Martinez

**Affiliations:** 1 University of South Florida Morsani College of Medicine, USF LVHN SELECT program, Tampa, Florida, USA; 2 Medicine, division of cardiology, Lehigh Valley Health Network, Allentown, Pennsylvania, USA; 3 Radiology, Lehigh Valley Health Network, Allentown, Pennsylvania, USA; 4 Medicine, division of statistics, Lehigh Valley Health Network, Allentown, Pennsylvania, USA

## Background

Genetic testing is commonly used in the management of patients with hypertrophic cardiomyopathy (HCM). Some studies suggest patients who test positive for known markers have a greater risk for adverse outcomes. In addition, the presence of late gadolinium enhancement (LGE) on cardiac MRI (cMRI) has also been associated with poorer outcomes and prognosis. Whether positive genetic testing is a predictor of LGE on cMRI is unknown.

## Methods

All patients referred to the Lehigh Valley Health Network HCM center who had received genetic testing between December 2009 and December 2012 and a cMRI were included. Clinical data (including associated symptoms) and imaging data were recorded for all patients. Image data was reviewed independently and blinded to clinical data. Characterization of LGE was defined as present or absent. LGE present on cMRI was further investigated and measured for mass and standardized to the mass of the left ventricle (LGEi). Genetic testing was considered positive if a mutation was identified or negative if no mutation was detected.

## Results

63 patients (mean age 51.4 ± 18 years; 42.9% male, gene positive 52.4%) were included in which 35 received cMRI. LGE was defined as present in 14 patients, 9 of which had received positive genetic testing. Fisher's exact test resulted in a p-value of 0.217. Sensitivity and specificity for positive genetic testing showing LGE on cMRI was 45% and 66.7% respectively with a PPV of 64.3% and NPV of 47.6%. In those patients who were positive for LGE, LGEi appears to be trending higher in patients with positive genetic testing compared to those with negative genetic testing 4.0 ± 3.1% and 3.5 ± 2.1% respectively (p = 0.378).

## Conclusions

There was no significant indicator that patients whom tested positive for genes known to cause HCM showed increased rates of LGE on cMRI. However, there was a higher prevalence of LGE in gene positive patients that may become significant in a study with a larger patient population.

## Funding

None.

